# Hybrid treatment for lower limb revascularization in a patient with vascular graft infection: a case report

**DOI:** 10.1590/1677-5449.202101911

**Published:** 2022-05-10

**Authors:** Eduardo Lichtenfels, Nilon Erling, Newton Roesch Aerts, Andre Silvestri Reitz da Costa

**Affiliations:** 1 Universidade Federal de Ciências da Saúde de Porto Alegre – UFCSPA, Porto Alegre, RS, Brasil.

**Keywords:** prosthesis-related infection, endovascular procedures, limb salvage

## Abstract

Patients with severe arterial obstructive disease and critical limb ischemia associated with vascular graft infection have elevated morbidity and mortality rates and are at high risk of limb loss. We present the case of a 76-year-old male patient with left lower limb critical ischemia and a femoropopliteal vascular graft infection. We used a hybrid treatment approach with an open surgical approach to the inguinal and popliteal regions and used the vascular prosthesis as endovascular access for direct recanalization of the superficial femoral artery, because the long occlusion and extensive calcification had frustrated initial attempts at endovascular treatment. After endovascular recanalization, the infected graft was removed. Used in conjunction with open surgery, advances in endovascular techniques and materials offer new solutions for patients when usual procedures fail.

## INTRODUCTION

Patients with severe arterial occlusive disease involving critical ischemia in the lower limbs and occlusive disease at multiple levels have increasingly benefited from hybrid procedures, i.e., use of endovascular techniques in conjunction with open surgery. Several hybrid techniques have been used successfully over the years, the most common being use of endovascular procedures to treat obstructions proximal to the aortoiliac segment in conjunction with infrainguinal bypass.^1^^-^7

However, there is a group of patients in whom endovascular procedures are unsuccessful because of difficulties obtaining vascular access or related to obstructions caused by extensive calcification of the vessel. In such patients, classic arterial bypass surgery may be a treatment option. For patients in whom a bypass constructed with a synthetic graft shows signs of infection and must be removed, it is necessary to employ complex procedures to avoid loss of the limb.

This case report was approved by the Ethics Committee at the Irmandade da Santa Casa de Misericórdia de Porto Alegre (ISCMPA), decision number 4.960.071.

## CASE DESCRIPTION

The patient was a 76-year-old male admitted to a vascular unit with critical ischemia of the left lower limb, with femoral pulse present and ulceration with central necrosis of the distal anterolateral aspect of the leg (Rutherford class 5). The patient’s previous medical history included myelofibrosis associated with splenomegaly, thrombocytopenia, and chronic anemia, hypertensive heart disease, and liver disease associated with esophageal varices.

**Figure 1:**
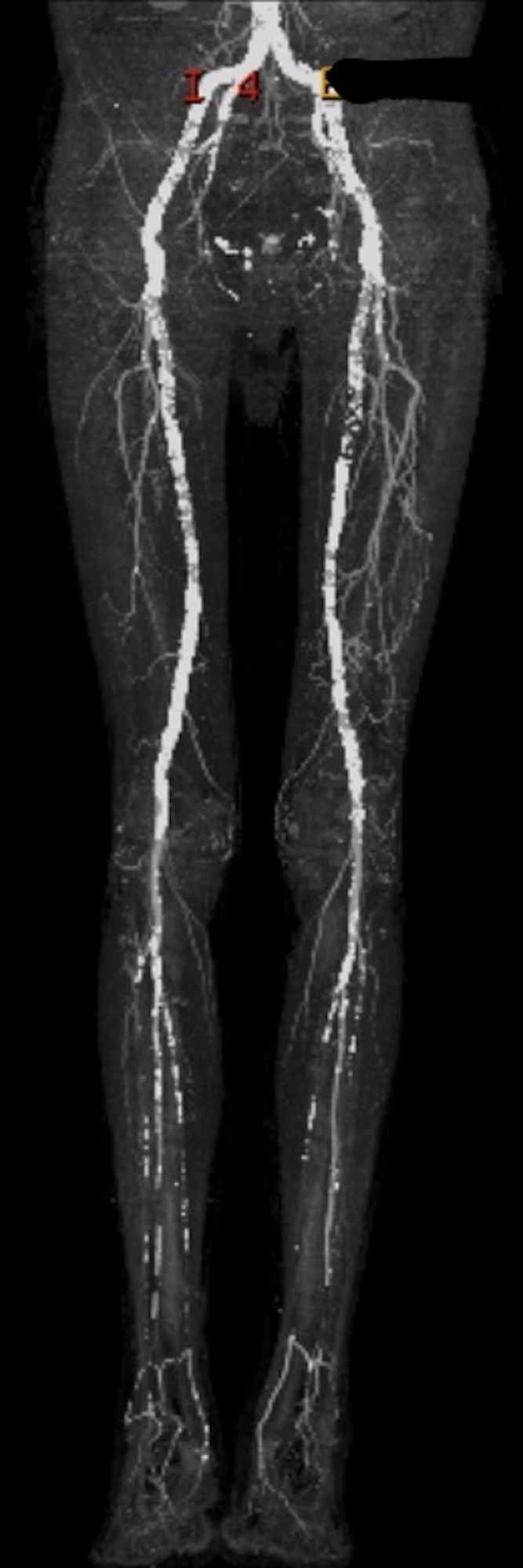
Preoperative angiotomography.

Preoperative angiotomography showed the aortoiliac segment with extensive calcifed plaques and luminal reduction less than 50%; patent common femoral artery with extensive calcifed plaques; superficial femoral artery with extensive calcifed plaques and proximal occlusion with recirculation through the proximal popliteal artery; patent popliteal artery with extensive calcifed plaques and areas of luminal reduction; patent tibioperoneal trunk with calcifed plaques and distal stenosis; patent fibular artery with proximal stenosis; and occluded tibial arteries (Figure 1).

The first attempt to employ endovascular treatment to recanalize the chronic occlusions of the superficial femoral artery and popliteal artery was unsuccessful. As the limb ischemia was progressing unfavorably, it was decided to conduct infrainguinal revascularization with a femoropopliteal bypass, constructed with a polytetrafluoroethylene (PTFE) graft because there was no adequate autologous graft material (vein). Endarterectomy of the common femoral artery was performed, because of the extensive circumferential calcification of the artery wall and presence of eccentric atherosclerotic plaques, combined with deep femoral arterioplasty. Proximal and distal flows were controlled using an embolectomy catheter because the calcifications prevented arterial clamping.

On the eighth postoperative day, the patient complained of discomfort in the left thigh, nausea, and lack of appetite and had leukocytosis and fever. The limb remained warm and femoral and popliteal pulses were present. Blood cultures were negative. Treatment with wide spectrum intravenous antimicrobials was started. Ultrasonography showed an anechoic collection around the graft in the proximal third of the thigh, measuring 3.0 x 0.7 cm and two other collections in the distal third of the thigh, measuring 2.3 x 1.5 cm and 1.8 x 1.3 cm. On the tenth postoperative day, the patient’s clinical status had worsened and he had significant signs of inflammation in the thigh.

**Figure 2:**
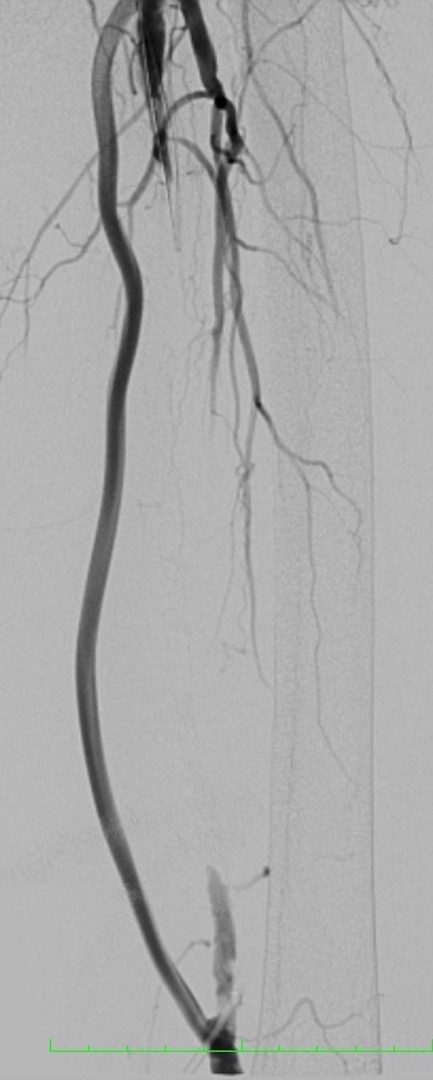
Preoperative angiography showing the bypass graft and the occluded superficial femoral artery.

On the 13th postoperative day, the infected graft was removed and the limb was revascularized using a two-stage hybrid approach: (1) endovascular recanalization of the native superficial femoral and popliteal arteries and (2) removal of the infected femoropopliteal graft and arterioplasty. The objective of treatment was to achieve sufficient distal arterial flow for the ulcer to heal and remove foci of infection. During this procedure, the arterial graft was punctured at the anastomosis with a 7F caliber introducer, both proximally and distally, avoiding puncture of the highly calcified native artery. Angiography demonstrated occlusion of the superficial femoral and popliteal arteries with recanalization above the joint line (Figure 2).

**Figure 3:**
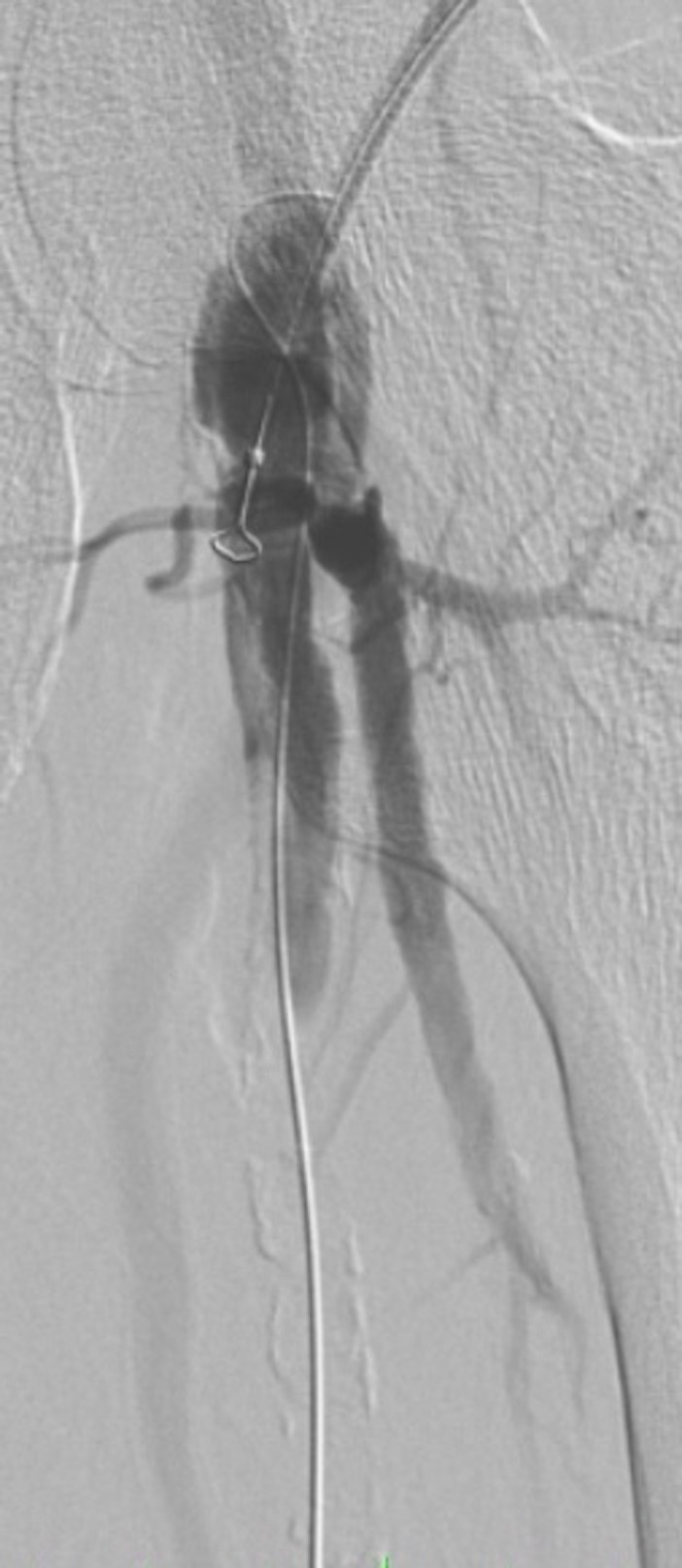
Angiography showing the anterograde accesses and the retrograde guidewire in the common femoral artery.

**Figure 4:**
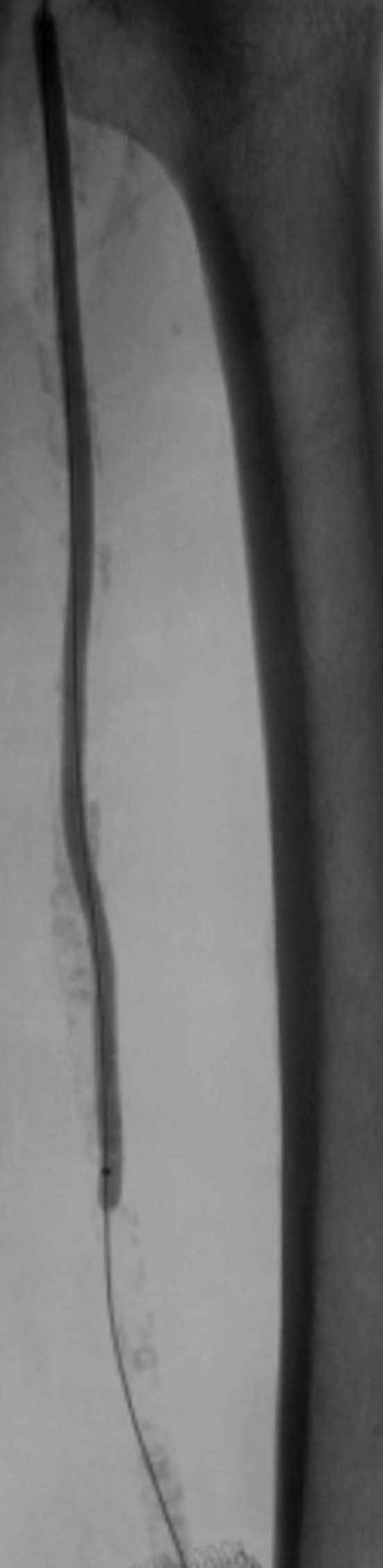
Angioplasty of the superficial femoral artery.

The obstruction was simultaneously recanalized via anterograde and retrograde approaches. Retrograde recanalization was achieved with a 300cm 0.018 guidewire and a 150cm 0.018 recanalization catheter, introduced via the distal superficial femoral access and removed via the common femoral access using a capture snare (Figure 3), followed by a through-and-through technique. Pre-dilation of the superficial femoral and popliteal arteries was achieved with a 4 x 200 mm Armada 18 balloon catheter (Abbott Vascular, Illinois, United States) (Figure 4). The proximal 7F introducer was then substituted for a 10F BRITE TIP introducer (Cardinal Health, Ohio, United States) and a 45 cm long 7F braided introducer was positioned in the popliteal artery below the anastomosis of the previous bypass. Selective popliteal angiography showed the popliteal artery patent, occlusion of anterior and posterior tibial arteries, subocclusive calcified plaque in the tibioperoneal trunk, and critical stenosis at the origin of the fibular artery. Infrapopliteal recanalization was achieved using a 0.018 guidewire. A 3 x 80 mm Pirouette balloon (Arravasc, Galway, Ireland) was then used to conduct angioplasty of the fibular artery and the tibioperoneal trunk. A 3.5 x 28 mm Multi-link balloon-expandable stent (Abbott Vascular) was used because of the significant recoil and dissection of the trunk lesion.

**Figure 5:**
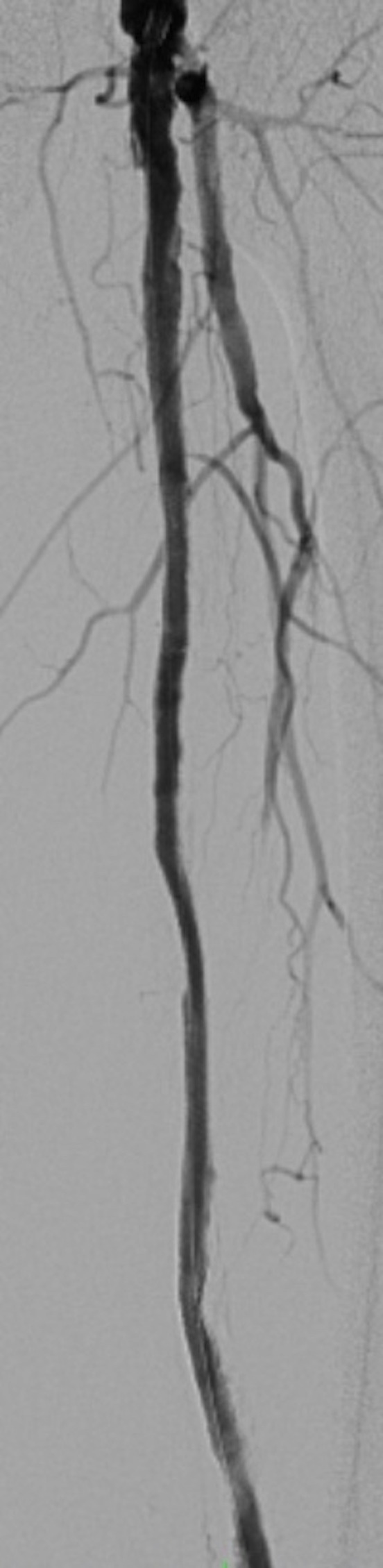
Post-procedural femoropopliteal angiography.

**Figure 6:**
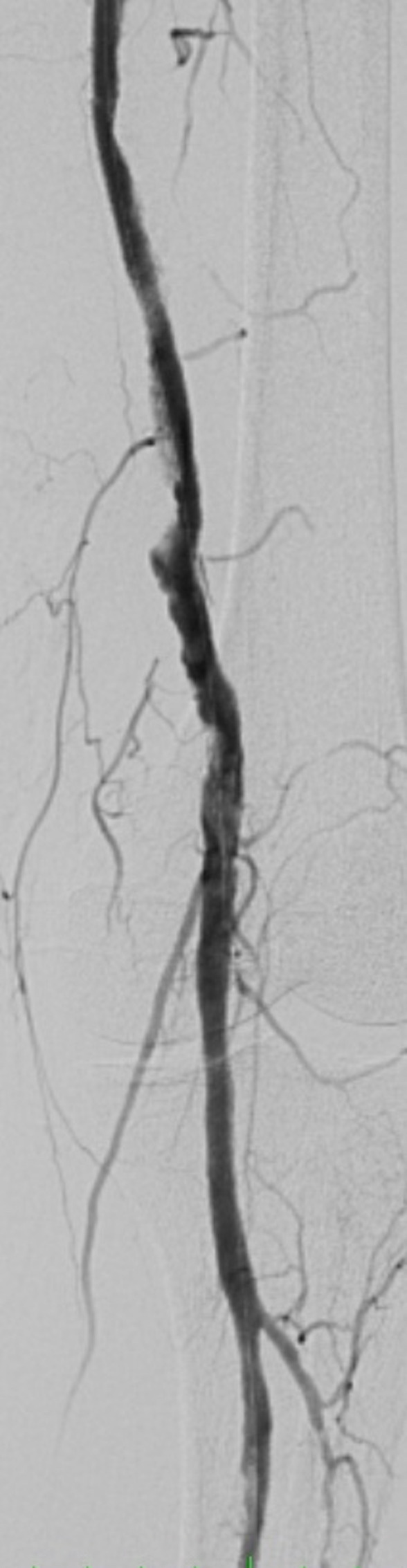
Post-procedural angiography of the distal popliteal artery.

Subsequently, the prosthesis was removed from the popliteal artery and arterioplasty was performed with a patch from the proximal segment of the saphenous vein of the thigh. Distal vascular control was obtained intraluminally with a 5 x 40 mm balloon catheter. Next, a 6 x 200 mm balloon catheter (Pirouette, Arravasc) was used to perform angioplasty of the popliteal artery and the superficial femoral artery via the femoral access, followed by deployment of a 5 x 180 mm Supera self-expanding stent (Abbott Vascular) in the popliteal artery, a 5x 150 mm Supera stent in the superficial femoral artery, and a 6 x 80 mm Absolute self-expanding stent (Abbott Vascular) in the proximal superficial femoral artery up to its ostium. The final angiography showed the treated segments were patent with good flow through the fibular artery (Figures 5 and 6). At the end of the procedure, the prosthetic graft was totally explanted and angioplasty of the common femoral artery was performed with a patch of the proximal saphenous vein from the thigh.

The patient recovered well and was discharged on the 14th postoperative day. At 20 months follow-up, the procedure was patent and the patient had a strong popliteal pulse and an ankle-brachial index of 0.8 and was free from trophic ulcers of the lower limb. The patient was followed-up with clinical examinations, measurement of ankle-brachial index, and Doppler ultrasonography without image recording.

## DISCUSSION

Combinations of endovascular procedures and open surgery are increasingly important for treatment of complex cases in patients with severe vascular disease involving several arterial levels and for complications of open surgery.^1^^-^7 The case described here illustrates the importance of a hybrid approach in a patient with critical lower limb ischemia, a failed initial endovascular procedure, and serious complications of the open surgery employed as an alternative.

One of the most important features of the procedure employed was use of sites proximal of the anastomoses from the first bypass as endovascular access, punctured under direct visualization, taking the opportunity afforded by the need for an open approach to remove the infected graft and thereby avoiding the need to puncture the highly calcifed native artery, which would have involved risk of dissection and arterial thrombosis. Another important feature that should be noted is the use of a retrograde popliteal access and an anterograde femoral access in conjunction to recanalize the occluded and extremely calcifed superficial femoral artery. Retrograde access via arteries of the leg or even puncture of the popliteal and femoral arteries is being adopted successfully for treatment of advanced disease involving the superficial femoral artery, as is puncture of occluded femoral stents. The success rate reported in the literature is 96-98.6%, with patency of 62.2-78% (for the period from 2006 to 2017), and low complication rates.^8^^-^10

Infection of the surgical site is a severe complication in vascular surgery, occurring in 0.9-22% of all arterial surgeries, while prosthetic vascular graft infection rates are in the range of 1.2-13%.^11^^-^15 Treatment is complex, involving multiple revisits, use of wide-spectrum antimicrobial, and prolonged hospital stays, resulting in elevated mortality (10-76%) and morbidity (8-53%).^11^^-^18

Extra-anatomic bypasses have been used for several decades as an option for revascularization of patients after removal of infected grafts in anatomic positions, as have native arteries endarterectomized by eversion, endarterectomized artery combined with veins, and antimicrobial-impregnated grafts.^11^^,^^14^^,^^17^ Another treatment option is to use the deep femoral vein for a new in situ bypass.^19^^,^^20^ Endovascular salvage of the native artery has been recommended as a treatment option for maintenance of arterial circulation in the limb, enabling the infected graft to be removed without harming the patient.^21^^,^^22^ One limitation we note is the fact that no more detailed imaging exams were employed to confirm patency of the procedure performed.

The case described illustrates the importance of the hybrid approach in a scenario in which endovascular techniques were unfeasible and open surgery had resulted in complications. The combination of several endovascular techniques with open surgery enabled resolution of a complex case and a favorable patient outcome.
