# Supplementary material for: Hybrid treatment for lower limb revascularization in a patient with vascular graft infection: a case report
Source: J Vasc Bras. 2022 May 10;21:e20210178. doi: 10.1590/1677-5449.202101911 (PMC9083548; doi:10.1590/1677-5449.202101911)
Supplement: Translation

# Translation: Tratamento híbrido para revascularização de membro inferior em paciente com infecção de prótese vascular: relato de caso

**DOI:** 10.1590/1677-5449.202101912

**Authors:** Eduardo Lichtenfels, Nilon Erling, Newton Roesch Aerts, Andre Silvestri Reitz da Costa

**Affiliations:** 1 Universidade Federal de Ciências da Saúde de Porto Alegre – UFCSPA, Porto Alegre, RS, Brasil.

**Keywords:** infecção de prótese, procedimentos endovasculares, salvamento de membro

## Abstract

Pacientes portadores de doença arterial obstrutiva periférica com isquemia crítica do membro associada a infecção de prótese vascular apresentam elevadas taxas morbimortalidade e alto risco de perda do membro. Apresentamos o caso de um paciente masculino de 76 anos com isquemia crítica do membro inferior esquerdo associada a infecção de prótese vascular femoropoplítea. Utilizamos abordagem híbrida para o tratamento com acesso cirúrgico das regiões inguinais e poplíteas, sendo a prótese vascular utilizada como acesso endovascular para recanalização direta da artéria femoral superficial devido a obstrução longa e extensa calcificação, que impediram as tentativas iniciais de tratamento endovascular. Após a recanalização endovascular, a prótese infectada foi retirada. O avanço das técnicas e materiais endovasculares em associação com a cirurgia aberta permitem novas soluções para pacientes quando os procedimentos habituais falham.

## INTRODUÇÃO

Os pacientes portadores de doença arterial obstrutiva periférica grave associada a isquemia crítica dos membros inferiores e doença oclusiva em múltiplos níveis têm se beneficiado cada vez mais dos procedimentos híbridos, ou seja, a associação de técnicas endovasculares com a cirurgia aberta. Várias técnicas híbridas têm sido utilizadas com sucesso ao longo dos anos, sendo a associação de procedimento endovascular para tratar obstruções proximais do setor aortoilíaco com a ponte infra-inguinal a mais frequente^1^^-^7.

Existe, no entanto, um grupo de pacientes em que o procedimento endovascular não obtém sucesso devido a dificuldades de acesso vascular ou associadas a obstruções com calcificações extensas do vaso. Nesses pacientes, a cirurgia clássica de ponte arterial pode ser uma opção terapêutica. Nos pacientes em que a ponte com prótese sintética apresenta sinais de infecção e precisa ser retirada, existe a necessidade de procedimentos complexos para evitar a perda do membro.

Este relato de caso foi aprovado pelo Comitê de Ética da Irmandade da Santa Casa de Misericórdia de Porto Alegre (ISCMPA), parecer número 4.960.071.

## DESCRIÇÃO DO CASO

Paciente masculino, 76 anos, admitido em unidade vascular com isquemia crítica de membro inferior esquerdo, pulso femoral presente e úlcera com necrose central na face anterolateral distal da perna (classe 5 de Rutherford). Na história médica pregressa, o paciente apresentava mielofibrose associada a esplenomegalia, plaquetopenia e anemia crônica, cardiopatia hipertensiva e hepatopatia associada a varizes esofágicas.

A angiotomografia pré-operatória demonstrou o segmento aortoíliaco com placas calcificadas extensas com redução luminal menor que 50%; artéria femoral comum pérvia com extensas placas calcificadas; artéria femoral superficial com extensas placas calcificadas e oclusão proximal com recirculação na artéria poplítea supragenicular; artéria poplítea pérvia com extensas placas calcificadas e áreas de redução luminal; tronco tibiofibular pérvio com placas calcificadas e estenose distal; artéria fibular pérvia com estenose proximal; e oclusão de artérias tibiais (Figura 1).

**Figura 1:** Angiotomografia pré-operatória.

A primeira tentativa de tratamento endovascular não obteve sucesso na recanalização das oclusões crônicas de artéria femoral superficial e artéria poplítea. Com a evolução desfavorável da isquemia do membro, optou-se pela revascularização infra-inguinal através de ponte femoropoplítea com prótese de politetrafluoretileno (PTFE), devido à ausência de enxerto autólogo (veia) adequado. Foi realizada endarterectomia da artéria femoral comum devido à extensa calcificação circunferencial da parede arterial e à presença de placas ateroscleróticas excêntricas, associada a arterioplastia femoral profunda. A interrupção dos fluxos proximal e distal foi obtida com cateter de embolectomia devido à impossibilidade de utilização de *clamp* arterial pelas calcificações.

No oitavo dia de pós-operatório, o paciente iniciou com desconforto na coxa esquerda, náusea, inapetência, leucocitose e febre. O membro permanecia aquecido e com pulsos femoral e poplíteo presentes. As hemoculturas solicitadas foram negativas. Foi iniciado tratamento com antimicrobianos intravenosos de amplo espectro. A ultrassonografia mostrou uma coleção anecoica periprótese no terço proximal da coxa medindo 3,0 x 0,7 cm e duas outras coleções no terço distal da coxa com 2,3 x 1,5 cm e 1,8 x 1,3 cm. No décimo dia de pós-operatório, o paciente apresentou piora do estado clínico e sinais flogísticos significativos na coxa.

No 13º dia de pós-operatório, a prótese infectada foi retirada, e o membro foi revascularizado por abordagem híbrida, em duas etapas: (1) recanalização endovascular das artérias femoral superficial e poplítea nativas e (2) retirada da prótese femoropoplítea infectada e plastia arterial. O objetivo do tratamento foi permitir um fluxo arterial distal adequado para que a úlcera cicatrizasse, bem como remover o foco infeccioso. Durante o procedimento, foi realizada punção da prótese arterial junto da anastomose com introdutor calibroso (7F), tanto proximal quanto distal, evitando a punção da artéria nativa muito calcificada. A angiografia demonstrou oclusão das artérias femoral superficial e poplítea com recanalização acima da interlinha articular (Figura 2).

**Figura 2:** Angiografia pré-operatória demonstrando a prótese/ponte e a artéria femoral superficial ocluída.

A obstrução foi recanalizada simultaneamente via anterógrada e retrógrada. A recanalização retrógrada foi obtida com fio guia 0,018, de 300 cm, e cateter de recanalização 0,018, de 150 cm, introduzidos pelo acesso femoral superficial distal, sendo exteriorizados pelo acesso femoral comum com a utilização de laço de captura (Figura 3) e posterior técnica em varal. Foi realizada pré-dilatação das artérias femoral superficial e poplítea com cateter balão 4 x 200 mm Armada 18 (Abbott Vascular, Illinois, EUA) (Figura 4). Posteriormente, o introdutor 7F proximal foi substituído por introdutor 10F BRITE TIP (Cardinal Health, Ohio, EUA), e um introdutor aramado longo 7F, de 45 cm, foi posicionado na artéria poplítea abaixo do ponto de anastomose da ponte prévia. A angiografia seletiva poplítea demonstrou perviedade da mesma, oclusão de artérias tibiais anterior e posterior, tronco tibiofibular com placa calcificada suboclusiva e estenose crítica na origem da artéria fibular. A recanalização infrapoplítea foi obtida com fio guia 0,018. Posteriormente, foi realizada angioplastia da artéria fibular e do tronco tibiofibular com balão 3 x 80 mm Pirouette (Arravasc, Galway, Irlanda). Devido a dissecção e *recoil* significativo da lesão do tronco, foi implantado stent balão-expansível 3,5 x 28 mm Multi-link (Abbott Vascular).

**Figura 3:** Angiografia demonstrando os acessos anterógrados e fio guia retrógrado na artéria femoral comum.

**Figura 4:** Angioplastia da artéria femoral superficial.

Posteriormente, a prótese foi retirada da artéria poplítea, e foi realizada arterioplastia com remendo de segmento proximal da veia safena da coxa. O controle vascular distal foi feito de forma intraluminal com cateter balão 5 x 40 mm. Em seguida, foi realizada a angioplastia da artéria poplítea e da artéria femoral superficial com cateter balão 6 x 200 mm (Pirouette, Arravasc) via femoral e implantados stent autoexpansível 5 x 180 mm Supera (Abbott Vascular) na artéria poplítea, stent 5x 150 mm Supera na artéria femoral superficial e stent autoexpansível 6 x 80 mm Absolute (Abbott Vascular) na artéria femoral superficial proximal até seu óstio. A angiografia final demonstrou perviedade dos segmentos tratados com bom fluxo pela artéria fibular (Figura 5 e 6). Ao final do procedimento, a prótese foi totalmente explantada, e foi realizada arterioplastia femoral comum com remendo de veia safena proximal da coxa.

**Figura 5:** Angiografia femoropoplítea pós-procedimento.

**Figura 6:** Angiografia poplíteo-distal pós-procedimento.

O paciente teve evolução favorável, recebendo alta no 14^o^ dia de pós-operatório. No seguimento de 20 meses, apresentava perviedade do procedimento com presença de pulso poplíteo amplo, índice tornozelo-braço 0,8 e ausência de lesões tróficas no membro inferior. O paciente foi acompanhado com exame clínico, medição do índice tornozelo-braço e eco-Doppler sem registro gráfico.

## DISCUSSÃO

A associação de procedimentos endovasculares com a cirurgia aberta vem ganhando importância no tratamento de casos complexos de pacientes portadores de vasculopatia grave acometendo vários níveis arteriais e em complicações da cirurgia aberta^1^^-^7. O caso apresentado demonstra a importância da abordagem híbrida em um paciente portador de isquemia crítica do membro inferior associada a falha do procedimento endovascular inicial e complicação grave da cirurgia aberta utilizada como alternativa.

Um dos aspectos fundamentais do procedimento realizado foi a utilização dos locais próximos das anastomoses da ponte prévia como acesso endovascular sob visão direta, aproveitando a necessidade de abordagem a céu aberto para a retirada da prótese infectada, evitando, assim, a punção da artéria nativa muito calcificada, com risco de dissecção e trombose arterial. Outro aspecto importante a ser salientado é a utilização do acesso retrógrado via poplítea e acesso anterógrado via femoral de forma concomitante na recanalização da artéria femoral superficial ocluída e extremamente calcificada. O acesso retrógrado através de artérias da perna ou mesmo da punção da artéria poplítea e femoral vem sendo utilizado com sucesso no tratamento da doença avançada da artéria femoral superficial, assim como a punção de stents femorais ocluídos. A taxa de sucesso reportada na literatura é de 96-98,6%, com perviedade de 62,2-78% (período de 2006 a 2017) e baixas taxas de complicação^8^^-^10.

A infecção do sítio cirúrgico é uma complicação grave da cirurgia vascular, ocorrendo em 0,9-22% de todas as cirurgias arteriais, sendo a taxa de infecção de prótese vascular de 1,2-13%^11^^-^15. O tratamento é complexo, envolvendo múltiplas reoperações, utilização de antimicrobianos de amplo espectro e tempo de internação prolongado, resultando em elevadas taxas de mortalidade (10-76%) e morbidade (8-53%)^11^^-^18.

A utilização de derivações extra-anatômicas como opção para revascularizar o paciente após retirada da prótese anatômica infectada vem sendo realizada há várias décadas, bem como a utilização de artéria nativa endarterectomizada por eversão, artéria endarterectomizada associada a veia e próteses impregnadas com antimicrobianos^11^^,^^14^^,^^17^. Outra opção terapêutica é o uso de veia femoral profunda para nova derivação *in situ* 19^,^^20^. O resgate endovascular da artéria nativa tem sido preconizado como uma alternativa terapêutica para a manutenção da circulação arterial do membro, possibilitando a retirada da prótese infectada sem prejuízo ao paciente^21^^,^^22^. Como limitação, citamos o fato de não ter sido realizado exame de imagem mais detalhado comprovando a perviedade do procedimento realizado.

O caso descrito ilustra a importância da abordagem híbrida em um cenário de limitação da técnica endovascular e de complicação da técnica aberta. A combinação de variadas técnicas endovasculares com cirurgia aberta possibilitou a resolução de um caso complexo e a evolução favorável do paciente.
